# Constructing public health policies in post crisis countries: lessons to learn from the associations between free-sugars consumption and diabetes, obesity and dental caries before, during and after sanctions in Iraq

**DOI:** 10.1007/s10389-016-0745-4

**Published:** 2016-06-18

**Authors:** E. Joury, R. Al-Kaabi, Anwar R. Tappuni

**Affiliations:** 1 Population and Patient Health, King’s College London Dental Institute, Denmark Hill Campus, Bessemer Road, London, SE5 9RS UK; 2 Institute of Dentistry, Barts and The London School of Medicine and Dentistry, Queen Mary University of London, Turner Street, London, E1 2AD UK; 3 Directorate of Preventive Health Affairs, Directorate General of Health, Duhok, Kurdistan Region Iraq

**Keywords:** Free-sugar consumption, Type-2 diabetes, Obesity, Dental caries, Non-communicable diseases (NCDs), Public health policy, Crisis

## Abstract

**Background:**

This article aims to provide evidence for an informed public health policy on free-sugar consumption in post-crisis countries.

**Methods:**

Iraq was selected as a case study. A systematic search for published data on the prevalence/incidence of type-2 diabetes, overweight/obesity, dental caries and free-sugar consumption levels in Iraq was conducted using MEDLINE, the Iraqi Academic Scientific journals and relevant international organisations’ websites. Comparable data before (1980–1990), during (1991–2002) and after (2003–2015) the United Nations sanctions (UNS) were included.

**Results:**

Ten studies were included. Quality scores ranged between 3 and 7/8. Free-sugar consumption decreased dramatically during the UNS (from 50 to 16.3 kg/person/year) and started increasing afterwards (24.1 kg/person/year). Changes in type-2 diabetes, overweight/obesity and caries levels mirrored those of free-sugar consumption. Caries declined markedly during UNS and started increasing afterwards. Comparable data on diabetes and overweight/obesity were only available for the periods during and after the UNS. Both of these conditions started increasing with increased free-sugar consumption after lifting the UNS.

**Conclusions:**

There is a need to develop a public health policy in post-crisis countries to maintain the reduction in free-sugar consumption, and hence promote both general and dental health, by integrating the common risk factor approach into the social determinant framework.

## Introduction

The Middle East region has recently faced a marked increase in the burden of free-sugar-related non-communicable diseases (NCDs), particularly type-2 diabetes, overweight/obesity and dental caries (Mirmiran et al. 2010; Boutayeb et al. 2012; Abid et al. 2015). Free sugars, a determinant and common risk factor across the aforementioned NCDs (Ruxton et al. 2010; Te Morenga et al. 2013; Moynihan and Kelly 2014), are defined as monosaccharides and disaccharides added to foods by the manufacturers, cooks or consumers and sugars naturally present in honey, syrups, fruit juices and fruit concentrates (World Health Organization and Food Agriculture Organization 2002). Currently, many countries in the Middle East are suffering from crises relate to political conflicts and economic sanctions. This is also relevant to all conflict zones in Africa, Asia and other parts of the world. These political, economic and social negative events have a profound impact on a population’s different living aspects, including diet and nutrition (Guha-Sapir and D’Aoust 2010). The latter undergo changes during crisis time, leading to both negative and positive changes in the population’s health and disease profile. For example, whilst the levels of mal- and under-nutrition increase dramatically (Guha-Sapir and D’Aoust 2010), free-sugar-related NCD levels are expected to decline markedly. This decline could be directly linked to the declining levels of free-sugar availability (Jamel et al. 2004) due to the disturbance in food channels (whether imported or locally produced) during the crisis time (Guha-Sapir and D’Aoust 2010). Yet, in post-crisis periods and with the recovery of the economics and increasing availability of free sugars, the levels of the abovementioned NCDs are expected to start increasing again.

Previous papers and reports have focused on discussing the health consequences of conflicts and wars and proposed post-crisis programmes pertaining to tackling the hunger, death and illness that emerge during sanctions or conflict times (Popal 2000; Jamel et al. 2004; Guha-Sapir and D’Aoust 2010). However, it is also crucial to plan for post-crisis programmes to prevent free-sugar-related NCDs, which are expected to start increasing when the population undergoes the usual nutritional transition post crisis. Thus, presenting a case study that analyses the association between free-sugar consumption and related NCDs before, during and after a crisis time is of paramount importance to develop evidence-based recommendations to inform governmental leaders and policy makers on potential public health policies and strategies that could maintain the low levels of free-sugar consumption and hence help control NCDs during the reconstruction time (Sheiham and Williams 2015). These policies and strategies should go hand in hand with other governmental efforts to improve public education and the deteriorated health and nutrition status of the population.

To the best of our knowledge, no previous study has assessed the association between changes in free-sugar consumption and changes in the levels of free-sugar-related NCDs, namely, type-2 diabetes, overweight/obesity and dental caries experiences before, during and after crisis time. Therefore, the current article aimed to: (1) review the published comparable data of a selected country pertaining to the free-sugar consumption and type-2 diabetes, overweight/obesity and dental caries prevalence and/or the incidence in both adults and children before, during and after the crisis time; (2) provide recommendations to help develop an informed evidence-based public health policy on free-sugar consumption in post-crisis countries.

## Methods

### Selecting a case study

Several countries in the Middle East are currently suffering from civil wars, political conflicts and economic sanctions. Iraq is a country in this region that has relatively recently gone through a crisis of economic sanctions and war and at present is considered to be in the post-crisis era. Therefore Iraq represents a good case for studying the changes in the health status of a population who have been through a crisis. The free-sugar-related NCDs in the Iraqi population in the past 30 years present an interesting model to study the effects of sugar availability on these diseases before, during and after crisis time.

Iraq has undergone a series of extreme political, social and economic changes due to wars and sanctions. In the 1970s, the economy was flourishing and the country was considered as one of the most advanced in the Middle East region. In 1980 Iraq entered an 8-year war with Iran, which lasted until 1988. During this period the situation inside Iraq remained relatively stable and prosperous. Health services remained reasonably well maintained and accessible (Diaz and Garfield 2003). In 1990, the World Bank categorised Iraq as one of the middle-income countries. There was a well-established public services infrastructure and extensive accessible health care. The Iraqi health system at the time was considered to be one of the best in the region. There was a low incidence of health problems related to deprivation or inappropriate lifestyles (Popal 2000).

In 1991, Iraq entered the Gulf War and the United Nations (UN) imposed sanctions, which lasted until 2003. During the period of 1991–1996, food and medicine imports were 85–90 % lower than before 1991 (Diaz and Garfield 2003). During this period the general health of the population deteriorated considerably and there was a significant increase in mortality rates (Diaz and Garfield 2003). The Iraqi health system was near collapse. The unemployment rate increased rapidly and the purchasing power decreased significantly, affecting the ability to buy nutritional food. The Government Food Ration scheme showed that the food energy (kcal) decreased by 65 % from 3120 in 1988/1990 to 1093 in the sanctions period. The daily intake of essential nutrients, such as calcium and iron, decreased by 83 % and 68 %, respectively (Popal 2000). Consequently, rickets, stunted growth and other vitamin-deficiency-related conditions were being reported (United Nations Economic and Social Development Department 1997).

In 1994, the Iraqi government reported that due to the sanctions it was unable to provide even the basic health needs of food and medicine to the population. This in turn put pressure on the UN to accept the Oil for Food Programme for the rest of the sanction period (1997–2003), which meant that more food and medicine were allowed into the country and consequently the general wellbeing of the population improved and the death rate declined (Diaz and Garfield 2003). Currently, according to the most recent World Health Organisation country profile for 2010 (World Health 2010a), NCDs including those related to free-sugar consumption were identified as a major cause of mortality in Iraq, accounting for 44 % of the deaths (World Health Organisation 2010b).

### The review search strategy

A systematic search was conducted to identify studies presenting the prevalence and/or incidence of type-2 diabetes, overweight/obesity or dental caries in Iraq, published between 1 January 1980 and 31 July 2015, in the English language.

Electronic searches were carried out in MEDLINE via Ovid and the Iraqi Academic Scientific journals. In MEDLINE, keyword- and MeSH-based searches were performed. The MeSH terms were “Diabetes Mellitus, type 2”, “obesity” and “dental caries”, and the keywords were “Iraq*”, “diabetes type 2”, “obesity/obese”, “dental caries” and “decay*”. The search strategy in the Iraqi Academic Scientific journals included the keywords “Iraq*”, “diabetes type 2”, “obesity”, “dental caries” and “decay*”. In addition, the following websites, data banks and archives were also searched: the World Health Organisation, UNICEF and the International Diabetes Federation. References were also identified from the retrieved articles and reports.

For sugar consumption data in Iraq, the publications of the International Sugar Organisation were searched.

### Study selection criteria

One trained reviewer (EJ) performed searches, selected studies, assessed publication validity and extracted the data. Prevalence/incidence studies that included hospital-based samples or samples of specific groups such as individuals with special needs or medical conditions were excluded. Studies that included caries severity with no report on prevalence were excluded. Studies with samples representing national or community populations were included if comparable data, in terms of the population’s socio-demographic characteristics (age and urban/rural residency), diagnostic criteria and the measure of quantification of disease existence or occurrence, were present across at least two different time periods.

An assessment of methodological quality was conducted using the critical appraisal guidelines for research articles reporting prevalence/incidence, developed by Loney et al. (1998). The scoring system consisted of eight dichotomous questions, with 1 for yes and 0 for no. The quality aspects covered the validity of the study design [appropriate sampling methods and frame, adequate sample size, appropriate outcomes measurements and assessment and response rates (>70 %)], interpretation (prevalence/incidence reported with confidence intervals) and applicability of the results (detailed description of study subjects and setting). All questions were weighted equally with higher scores indicating better methodological quality of the studies.

### Data extraction

The following data were extracted from the included studies/reports: study time, population (national/community, age, urban/rural residency), sample size and disease prevalence/incidence. For dental caries, data on disease severity as measured by dmft/DMFT^1^ were also extracted. If more than one study was included for any specific disease in a particular time period, data were extracted from the study with the higher quality score.

## Results

**Fig. 1:**
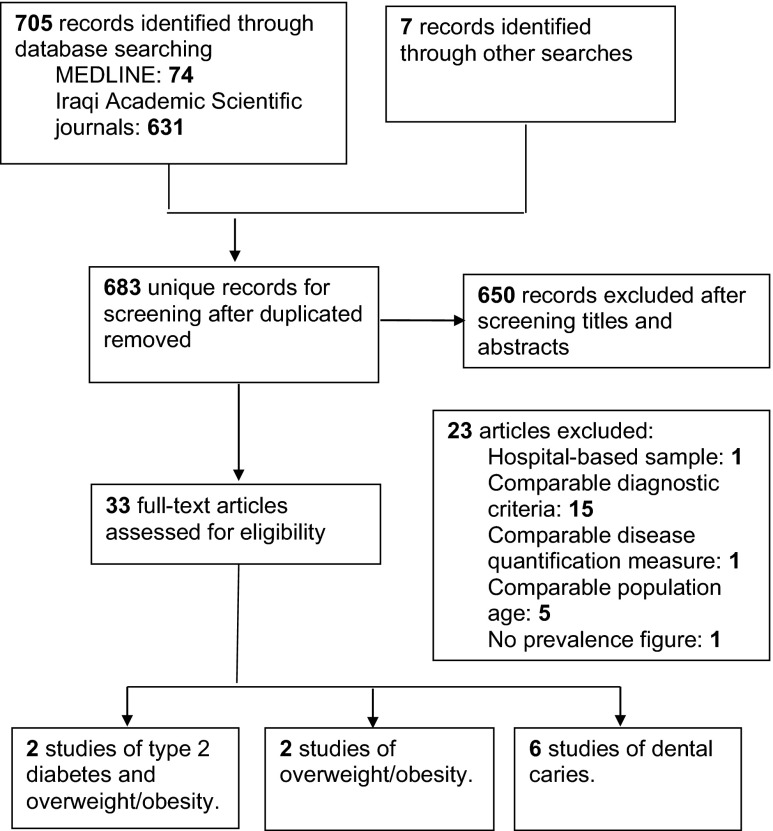
Flowchart of the selection of studies for the review

A flowchart describing the review search results is presented in Fig. 1. Overall ten studies were included in the current review, two national studies conducted by the Iraqi Ministry of Health in collaboration with the World Health Organisation in 2000 and 2006, which included data on the prevalence of type-2 diabetes and overweight/obesity among adults, two community-based studies conducted in 2002 and 2010/2011 on the prevalence of overweight/obesity among children and six community-based studies carried out on the prevalence of dental caries among children (Table 1).

**Table 1:** Summary of studies included in the review

Reference	Study time	Population (sample size)	Disease	Quality score/8
Al-Ani and El-Samarrai 2014	After UN sanctions	Urban community of 12-year-old school children in Heet city (764)	Dental caries	6
Alwan et al. 2009	After UN sanctions, 2006	Urban community of 9–12-year-old school children in Baghdad city (400)	Dental caries	4
Hassan and Al-Taai 2006	After UN sanctions, 2004–2005	Urban community of 5-year-old kindergarten children in Baghdad city (569)	Dental caries	4
Jamel et al. 1997	Before UN sanctions, 1986	Urban and rural community of 6–7-, 11–12- and 14–15-year-old school children in Baghdad governorate (3,002)	Dental caries	6
Jamel et al. 2004	Before and during UN sanctions, 1985; 1995	Urban and rural community of 6–7-, 11–12- and 14–15-year-old school children in Baghdad governorate (3,015)	Dental caries	7
Lafta and Kadim 2005	During UN sanctions, 2002	Urban and rural community of 7–13-year-old school children in Babil governorate (8,300)	Overweight/obesity	6
Mughamis and Mohammed 2014	After UN sanctions, 2013	Urban and rural community of 15-year-old school children in Maysan governorate (750)	Dental caries	3
Salman and Ajeel 2013	After UN sanctions, 2010–2011	Urban community of 6–15-year-old school children in Basrah governorate (1,466)	Overweight/obesity	4
Ministry of Health 2003	During UN sanctions, 2000	National survey for 25–65-year-old adults (13,430)	Overweight/obesity and type-2 diabetes	7
Ministry of Health 2006	After UN sanctions, 2006	National survey for 25–65-year-old adults (9,345)	Overweight/obesity and type-2 diabetes	7

The quality scores of included studies ranged between 3 and 7 (Table 1). The major quality flaws identified were related to the potential bias in the disease assessment (not reporting examiner’s training and calibration or examiner’s reliability), the inadequacy of sample size (including not mentioning the sample size calculation assumptions) (n = 4), not reporting the response rate (n = 4), not presenting the 95 % confidence interval for the disease prevalence (n = 5), the non-random sampling selection (including consecutive or non-random selection in the second stage of multistage sampling) (n = 3) and the absence of a detailed description of subjects and settings (n = 3).

### Prevalence of type-2 diabetes, overweight/obesity and dental caries

Comparable data on type-2 diabetes and overweight/obesity levels in the Iraqi population were available for the periods during and after the UNS for adults aged 25–65 years (Ministry of Heath 2003; 2006) and children aged 6–13 years (Lafta and Kadim 2005; Salman and Ajeel 2013) (Tables 1 and 2). These data showed a marked increase in type-2 diabetes and overweight/obesity prevalence after the UNS.

**Table 2:** Free-sugar consumption, type-2 diabetes, overweight/obesity and dental caries levels in the Iraqi population before, during and after United Nations (UN) sanctions

Variable	Area	Before UN sanctions	During UN sanctions	After UN sanctions
Free-sugar consumption kg/person/year	–	50	16 · 3	24.1
Type-2 diabetes^a^ Prevalence in adults	–	NA*	4.1 %	6.5 %
Overweight/obesity				
Prevalence in adults aged 25–65 years, overweight, obesity^a^	–	NA* NA*	5.5 % NA*	34.1 % 32.8 %
Prevalence in children aged 6–13 years, overweight obesity^b,c^	Urban	NA* NA*	6 % 1.3 %	13.6 % 10.5 %
Dental caries^				
**5–6 year olds** ^**d,e**^ Prevalence (mean dmft)	Urban	80.5 % (7.6)	61.2 % (3.8)	NA*
	Rural	36.5 % (1.9)	7.1 % (0.7)	18.8 % (5.1)
**11–12 year olds** ^**d,f**^ Prevalence (mean DMFT)	Urban	82.6 % (7.0)	66.1 (2.7)	90.2 % (NC**)
	Rural	39.1 % (1.3)	12.3 % (0.5)	NA*
**14–15 year olds** ^**d.g**^ Prevalence (mean DMFT)	Urban	87.7 % (8.3)	69.9 % (2.8)	90 % (NC**)
	Rural	41.2 % (1.8)	15.4 % (0.9)	96.3 % (NC**)

*NA, not available

**NC, not comparable as it was measured by DMFS

^All the selected dental caries data were collected using the World Health Organisation (1987) criteria

^a^Based on two national surveys (Ministry of Health 2003; 2006)

^b^Based on the Lafta and Kadhim study (2005)

^c^Based on the Salman and Ajeel study (2013)

^d^Based on the Jamel et al. study (2004)

^e^Based on the Hassan and Al-Taai study (2006)

^f^Based on the Al-Ani and El-Samarrai study (2014)

^g^Based on the Mughamis and Mohammed study (2014)

With respect to dental caries in the Iraqi population, comparable data were only available on the prevalence in children (Tables 1 and 2) (Jamel et al. 2004; Hassan and Al-Taai 2006; Alwan et al. 2009; Al-Ani and El-Samarrai 2014; Mughamis and Mohammed 2014). There was a marked decline in dental caries prevalence and severity (dmft/DMFT) across all child age groups over a 5-year period during the sanctions. Dental caries prevalence and severity were less than half that of the pre-sanction period in all child age groups (Table 2). After the UNS, the prevalence of dental caries started increasing, currently reaching a level higher than that before the UNS.

### Sugar consumption

The levels of sugar consumption in Iraq have fluctuated over the last 30 years. First, free-sugar consumption was 50 kg/person/year in the 1984–1990 period, according to the Iraqi Ministry of Trade 1990 (International Sugar Organisation 1984). Then, after the UNS in 1991, the free-sugar consumption dropped to an overall average of 16 · 3 kg/person/year until 2003 (International Sugar Organisation 2013). Subsequently, there was an increase in sugar consumption again after the sanctions were lifted in 2004 to 24 · 1 kg/person/year (International Sugar Organisation 2013).

## Discussion

This is the first study to present the association between free-sugar consumption and the levels of three NCDs, namely, type-2 diabetes, overweight/obesity and dental caries before, during and after a crisis time. Current recommendations include the creative use of existing data to piece together information from different sources to allow a better understanding of the dynamics of health needs in crisis-affected populations (Guha-Sapir and D’Aoust 2010). Changes in the levels of the aforementioned NCDs mirrored those of free-sugar consumption. The findings of the current review are in agreement with the findings of previous dental studies that investigated the association between free-sugar consumption and dental caries before, during and after the Second World War (Sognnaes 1948; Schulerud 1950; Takeuchi 1961). For example, in Japan, the dramatic decrease in dental caries during war time, and the subsequent increase after the war period, had mirrored the change of per capita free-sugar consumption levels, which decreased from 15 to 0.2 kg per year and then increased again to 15 kg per year over an 11-year period post war (Takeuchi 1961).

Systematic reviews have largely identified consistent evidence supporting a relationship between the amount of sugar intake and the development of type-2 diabetes (Ruxton et al. 2010), overweight and obesity (Te Morenga et al. 2013), and dental caries (Moynihan and Kelly 2014; Sheiham and James 2014a) across different age groups. For every increase of 150 kcal sugar/person/day, there is a 1.1 % increased prevalence of type-2 diabetes. In addition, for each 25 g per day sugar increase, one tooth per child would become carious (World Health Organization 2014).

The current study findings underpin the assertion that tackling the burden of free-sugar-related NDCs is only possible by focusing on strategies to limit free-sugar consumption. All available evidence showed clearly that the burden of free-sugar-related NCDs cannot be tackled using the traditional “Western-style” preventive and curative approaches, which are based on health education and treatment (Williams et al. 2015). These approaches were deemed to be unaffordable (exceeding available financial and human resources), unsustainable and ineffective (Sheiham and Williams 2015). Thus, post-crisis countries would benefit from focusing on effective strategies to maintain the low levels of free-sugar consumption rather than adopting the traditional preventive and curative approaches. Such strategies should target the broader upstream social factors that affect free-sugar consumption. The decision made by individuals to consume free sugars is deeply rooted in the social, economic and environmental conditions under which people grow, live, work and age. Thus, post-crisis countries should create sustainable health-promoting environments where health compromising behaviours, such as free-sugar consumption, are the difficult choice and health-conducive behaviours, such as vegetable and fruit consumption, are the easy choice, in terms of availability, accessibility, affordability and appropriateness.

Public health policies and the related regulations and legislations are the main means for creating sustainable health promoting environments. One way of achieving this is to first formulate country-specific goals to maintain the low levels of free-sugar consumption close to the latest recommended maximum of 5 % of total energy (E) (World Health Organization 2014). This equates to approximately 25 g/day for adults and children aged 7 years old and above, 22 g/day for 4–6 year olds and 16 g/day for 1–3-year-old children (Moynihan and Kelly 2014). The next step is to adopt the Health in All Policies (HiAP) framework, which will aid in developing intersectoral partnership among government ministries. This, in turn will yield, for post-crisis countries, better health for the whole population and large future financial benefits from preventing the growing unaffordable burden of NCDs.

The HiAP approach should include: introducing taxes on sugary foods and drinks (whether imported or locally manufactured), regulations and legislations pertaining to the food industry, policies on food provided in schools, nurseries and other public catering outlets, and restrictions on the advertisement of sugary foods and drinks (Sheiham and Williams 2015; Williams et al. 2015).

Sugar is easy to tax because it goes through a long chain. Taxes at the level of the processor are invisible, more acceptable and easier. The simplest form of free-sugar taxing is to introduce a tax on sugar as a mass commodity (Sheiham and James 2014b). Other taxing strategies, such as taxing individual sugary food or drink items based on their sugar content, involve extremely complex administrative processes. Experts suggest an increase by at least 20 % in the retail price of sugary items to achieve a desirable effect on consumers’ demand and hence consumption (Sheiham and James 2014b). At the same time no taxes are imposed on sugar-free foods, drinks or medicine products. Tax returns could be used to subsidise the prices of vegetables, fruits and sugar-free medicines.

The government should put pressure on the food and pharmaceutical industry, supported by appropriate legislations, to reduce the sugar content in their products and offer a wide range of sugar-free alternatives (Sheiham and James 2014b). Clear and unbiased labelling is essential. Products containing more than 2.5 % free sugars should be labelled as “high”.

Packed lunches and food provided in schools, nurseries and other public catering outlets, such as hospitals and nursing/care homes, should be sugar free or at least sugar reduced (Sheiham and James 2014b). Fruit juices and sugar-containing treats and confectionaries should be limited to a maximum of 2.5 % energy intake.

The post-crisis government should also set more stringent codes of practise and restrictions on the advertisement of sugary foods and drinks, particularly to children.

All the above-mentioned policies should be supported with initiatives to increase the public awareness of the need to reduce the intake of sugary food and drinks from infancy throughout the life course, using national nutrition and maternal and child programmes.

The current review is not without limitations. The main limitation of the present review pertains to the potential ecological fallacy. The latter implies a logical fallacy in the interpretation of statistical data where inferences about the nature of individuals are deduced from inference for the group to which those individuals belong (Greenland and Robins 1994). In other words, the reported levels of sugar consumptions cannot be links directly at an individual level to the reported levels of NCDs. A second limitation of the current review is related to the unavailability of some of the data and the low quality of some of the others. Some data, in particular time periods, were not available, such as the prevalence of type-2 diabetes and overweight/obesity before the UNS. In addition, one of the included studies had a quality score of 3, which is below the conventionally accepted score of 4. A third limitation of the present study pertains to the possibility of alternative explanations to the observed associations. For example, overweight/obesity and physical activity are part of type-2 diabetes etiological factors. Overweight and obesity, in turn, are determined by dietary intake and physical activity. The latter are likely also to co-vary across the three time periods, before, during and after UNS. The present review attempted to address such potential confounding; however, the absence of related comparable figures on physical activity in two time periods (before and during UNS) did not allow exploring this possibility. A final limitation of the current review relates to being conducted by only one trained reviewer.

It is worth mentioning that the current manuscript did not intend by any means to suggest that the negative effect of malnutrition can be balanced by the positive effect of lower sugar consumption during crisis time. The core intention is to highlight potential aspects that post-crisis governments can maintain whilst restoring other aspects influencing their populations’ health status and wellbeing.

## Conclusion

The case study presented in this article provides evidence for a need to develop a public health policy in post-crisis countries to maintain the reduction in free-sugar consumption that occurred during the crisis time to possibly maintain the low levels of related NCDs. Such a policy is likely to promote both general and dental health effectively and affordably by integrating the common risk factor approach into the social determinant framework to reduce the incidence of sugar-related NCDs.

## Footnotes

1 The dmft is an index to measure caries experience in primary dentition and it is expressed as the total number of decay (d), missing (m) and filled (f) deciduous teeth. The DMFT is an index to measure caries experience in permanent dentition and it is expressed as the total number of decay (D), missing (M) and filled (F) permanent teeth.
